# Expanding the identification of key resource combinations for mid- to long-term growth in electric vehicle market entry

**DOI:** 10.1371/journal.pone.0328563

**Published:** 2025-08-07

**Authors:** Min-je Cho, Kukjin Bae, Jeongeun Byun, Juneseuk Shin

**Affiliations:** 1 Center for Technology Commercialization Research, Korea Institute of Science and Technology Information, Dongdaemun-gu, Seoul, Republic of Korea; 2 Department of Systems Management Engineering, Sungkyunkwan University, Seobu-Ro, Jangan-gu, Suwon, Gyeonggi-do, Republic of Korea; Sunway University, MALAYSIA

## Abstract

This study examines the key resource combinations influencing electric vehicle (EV) adoption, differentiating between short-term market entry and mid- to long-term growth, using the Technology-Organization-Environment (TOE) framework and the Resource-Based View (RBV). Analyzing 12 companies from 2012 to 2022, we find that firms with a well-balanced combination of technological capabilities, organizational strategies, and environmental adaptability achieve sustained market diffusion. A deficiency in any one of these areas hinders long-term success, and excellence in a single resource alone is insufficient. Moreover, the resource requirements for initial market entry differ from those needed for sustained growth, highlighting the need for firms to dynamically adjust their resource strategies based on evolving technological advancements, organizational capabilities, and regulatory and market conditions. Theoretically, this study integrates innovation diffusion theory, RBV, and TOE to offer a comprehensive perspective on EV market dynamics. From a managerial standpoint, companies must develop technological advancements, organizational capabilities, and environmental adaptability to ensure long-term success. Policy-wise, governments can accelerate EV adoption by implementing targeted infrastructure investments, standardizing digital and charging networks, and supporting sustainable innovation through incentives and regulatory frameworks.

## 1. Introduction

Electric vehicles (EVs) are expected to play a significant role in future mobility due to their environmental and economic benefits [1]. In response to climate change, countries are expanding policies to promote and develop eco-friendly vehicles. As EV technology advances, it enhances convenience and value compared to traditional internal combustion engine vehicles [2]. Consequently, these government policies and technological advancements have rapidly propelled the EV industry over the past decade.

In the early stage, the EV market was primarily at the level of demonstration and vehicle validation. The number of companies releasing EV models began to increase after 2011, and it was not until 2017 that global EV sales exceeded 1 million units annually [3]. At this stage, a few companies like Tesla established themselves as market leaders, but most entrants failed to penetrate the automotive industry owing to entry barriers [4,5]. Entry barriers increased the entry costs for new entrants compared to incumbents, thus hindering their entry and limiting competition [5,6].From 2017 to 2022, EV sales surged more than tenfold from about 1 million to over 10 million units [7]. In the mid- to long-term diffusion and growth phase of EVs, numerous automotive OEM(Original Equipment Manufacturer)s also launched various EV products, accelerating market diffusion. Concurrently, the growth of EV companies was evident. Notably, Tesla achieved a 17% market share of all EVs sold worldwide in 2022, with a 40% increase in sales compared to the previous year.

To determine the main factors influencing this phenomenon, previous studies commonly agree that the spread of EVs is determined by conventional consumer perspective factors such as EV prices, total maintenance costs, driving range, and policies influencing these factors such as subsidies and tax reductions [8,9]. [4] identified the combination of key resources and factors that new entrants need to overcome entry barriers to the EV market entry phase. Companies like Ford, Nissan, Chevrolet, and Renault, which successfully entered the EV market, secured and developed key resources such as battery price competitiveness, vehicle performance, vehicle price, driving range, and safety and regulation compliance, utilizing external environmental changes in technology, market, and policy.

In this study, we apply the Technology-Organization-Environment (TOE) framework to analyze the determinants of EV market success for Mid- to Long-term Growth. The TOE framework provides a comprehensive and structured approach to understanding how firms adopt and integrate emerging technologies within their business strategies by considering technological capabilities, organizational dynamics, and external environmental influences [10]. Unlike previous research that primarily focused on consumer-based adoption factors, TOE enables us to examine the firm-level strategic factors that contribute to long-term EV market competitiveness [11].

The EV industry is characterized by rapid technological advancements, evolving regulatory landscapes, and complex supply chain dynamics, making it essential to analyze market success from multiple dimensions rather than through a single factor such as consumer preference [12]. TOE allows us to systematically explore how technological (battery advancements, charging infrastructure, vehicle efficiency), organizational (R&D investment, supply chain integration, production scalability), and environmental (government subsidies, regulatory policies, charging infrastructure expansion) factors interact to shape both market entry and long-term growth trajectories [13].

Furthermore, TOE is particularly suitable for studying the mid- to long-term evolution of the EV market, as it captures the dynamic nature of resource allocation and adaptation strategies required for sustainable industry expansion [14]. In the initial market entry phase, resources affecting cost reduction—such as battery and vehicle technology, supply chain capabilities, and simple EV architecture—had a short-term impact on the spread of EVs [15]. However, in the mid- to long-term growth phase, the factors influencing EV adoption became more diverse and complex, necessitating a holistic framework like TOE to capture these evolving dynamics [16].

Not only battery, charging, and vehicle technology but also brand reputation, infrastructure scale (charging time and convenience), advanced research and development (investment in next-generation batteries), regulatory compliance, and global production and logistics networks (low-cost, just-in-time supply) have been suggested as key resources influencing long-term market diffusion [12]. The TOE framework helps us categorize and analyze these factors within an integrated framework, ensuring that we account for technological readiness, organizational capabilities, and environmental enablers that drive sustained EV market success [17].

This study adopts the TOE framework to systematically examine how EV companies can leverage their technological capabilities, organizational strengths, and external environmental conditions to achieve long-term success. To enhance analytical clarity, we differentiate between key resources that directly influence sales and key adoption factors that mediate this relationship. Given the complexity of many-to-many relationships and data limitations, directly analyzing the impact of resources on key adoption factors presents significant challenges.

As a methodological approach, we first identify critical resources that drive sales performance and then assess the mediating role of key adoption factors. This study employs fuzzy-set qualitative comparative analysis (fs/QCA), which is particularly effective for handling a limited number of cases compared to traditional statistical methods. Since only 17 companies have entered the EV market, obtaining significant results through other quantitative techniques is challenging [18]. fs/QCA is well-suited for identifying combinations of independent variables that contribute to the causal relationship between independent and dependent variables, even with a small or moderate sample size [19].

Through this, this study aims to discern the combination of key resources and mid- to long-term growth factors in the EV market to help companies grow successfully. Additionally, this study strives to find the minimum threshold values of key capabilities and resources to avoid failures in market growth. Our research can improve the competitive strategies of automotive OEMs and further design long-term eco-friendly mobility growth policies through targeted resource subsidies and key capability enhancement programs linked to key resources in the EV industry.

The remainder of this paper is organized as follows. Section 2 reviews the literature on the Technology-Organization-Environment (TOE) framework and the Resource-Based View (RBV). Section 3 introduces our methodology, including the research framework, and Section 4 provides the analysis results. The discussion and conclusion follow, including technical, strategic, and policy implications.

## 2. Literature review

### 2.1. Technology-Organization-Environment (TOE) Framework

The Technology-Organization-Environment (TOE) framework provides a structured approach to understanding how firms adopt and integrate technological innovations by considering three key dimensions: technological capability, organizational factors, and environmental influences [18]. Unlike consumer-oriented models such as the Innovation Diffusion Theory (IDT), which primarily focuses on individual adoption behavior, the TOE framework emphasizes strategic decision-making at the firm level in leveraging internal resources and external conditions for market competitiveness [10]. Given the rapid evolution of the electric vehicle (EV) industry, TOE serves as a robust framework for analyzing the drivers of EV market growth and industry transformation [20].

The technology dimension within the TOE framework encompasses key innovations that determine the feasibility and attractiveness of EV adoption. Battery technology remains a fundamental factor, as improvements in energy density, charging speed, and overall cost reduction directly impact vehicle performance and affordability [21]. Similarly, the availability and efficiency of charging infrastructure play a critical role in shaping consumer confidence and large-scale market penetration [7]. Furthermore, advancements in vehicle efficiency, including powertrain optimization, aerodynamics, and AI-driven energy management, contribute to the increasing appeal of EVs as viable alternatives to traditional internal combustion engine vehicles [22]. These technological elements collectively define the competitive landscape for EV manufacturers and influence adoption rates across different market segments.

The organizational dimension focuses on the internal capabilities that firms must develop to successfully integrate EV technology into their business models. A well-structured supply chain is essential for maintaining cost-effective production, ensuring the availability of critical raw materials, and optimizing logistics networks [23]. Investments in research and development (R&D) are equally crucial, as firms that lead in battery innovation, software integration, and predictive maintenance systems gain a competitive edge in the market [21]. Additionally, brand strength and market positioning influence consumer trust and resale value, further reinforcing the long-term success of EV manufacturers [7]. Companies that effectively leverage these organizational resources are better positioned to scale production, enhance product differentiation, and achieve sustainable profitability.

The environmental dimension captures the external factors that influence EV market growth, including government policies, regulatory compliance, and infrastructure development. Many governments worldwide have introduced incentives such as tax exemptions, purchase subsidies, and carbon credit policies to accelerate EV adoption [9]. At the same time, regulatory frameworks related to emission reduction targets, safety standards, and battery recycling mandates create both challenges and opportunities for firms operating in the EV industry [24]. Beyond government policies, the expansion of charging infrastructure through public-private partnerships and smart grid integration is a key determinant of the long-term viability of EVs [21]. These external conditions not only shape market demand but also dictate the strategic direction of firms looking to enter or expand within the EV sector.

The EV industry has evolved through distinct adoption phases, requiring firms to continuously adapt their strategic priorities. In the early market entry phase, success was largely driven by cost reduction strategies, with a focus on battery price minimization, supply chain optimization, and modular EV architectures to improve affordability and scalability [8]. As the market transitions to the mid- and long-term growth phase, firms must prioritize competitive differentiation through brand reputation, regulatory compliance, next-generation battery investment, and global production scalability [25]. While cost efficiency remains important, the ability to innovate and adapt to regulatory and market pressures has become increasingly vital for long-term success.

Table 1 categorizes the key adoption factors under the TOE framework, highlighting the interplay between technology, organizational resources, and environmental influences in shaping the future of the EV industry.

**Table 1:** TOE Framework and Key Resources for EV Market Growth.

EV Adoption Factor	TOE Category	Key Resources & Capabilities	Time Phase	Studies
**Battery Price**	Technology (T)	R&D intensity, production scale efficiency, supplier relationships	Short-term	[22,26]
**Drive Range**	Technology (T)	Battery advancements, energy density improvements, vehicle optimization	Short-term	[8] [7,27]
**Charging Time & Options**	Technology (T)	Fast-charging R&D, charging port standardization, infrastructure compatibility	Mid- to Long-term	[7] [28,24]
**Battery Life & Sustainability**	Technology (T)	Advanced material R&D, thermal management, energy recycling technology, second-life battery applications	Mid- to Long-term	[25] [29]
**Vehicle Efficiency & Performance**	Technology (T)	Powertrain efficiency, aerodynamics, software optimization, energy consumption optimization	Mid- to Long-term	[30] [31]
**R&D Investment & Innovation**	Technology (T)	Investment in next-generation batteries, AI-driven mobility, smart energy management	Mid- to Long-term	[32]
**Vehicle Price & Cost Competitiveness**	Organization (O)	Cost structure optimization, supply chain efficiency, multi-brand platform sharing, localized production	Short-term	[33]
**Supply Chain & Production Scalability**	Organization (O)	Global supplier network, just-in-time logistics, flexible production capabilities	Mid- to Long-term	[34]
**Brand Strength & Resale Value**	Organization (O)	Brand perception, historical reliability, secondary market growth, end-user trust	Mid- to Long-term	[35]
**Infrastructure Scale & Convenience**	Environment (E)	Charging infrastructure expansion, ultra-fast charging technology, smart-grid integration, accessibility	Mid- to Long-term	[36]
**Vehicle Maintenance & Lifecycle Costs**	Organization (O)	Manufacturing process optimization, reliability engineering, after-sales service networks, predictive maintenance	Mid- to Long-term	[37]
**End-to-End Digital Integration**	Organization (O)	Supply chain integration, modular platform strategy, over-the-air (OTA) updates, autonomous driving readiness	Mid- to Long-term	[38]
**Government Incentives & Policy Support**	Environment (E)	Government subsidies, import/export tax exemptions, carbon credit markets, green energy transition policies	Short-term	[29]
**Regulatory Compliance & Global Logistics**	Environment (E)	Safety compliance, emission regulations, recycling policies, global trade policies, cross-border logistics	Mid- to Long-term	[39]
**Battery Recycling & Circular Economy**	Environment (E)	Circular economy policies, second-life battery utilization, closed-loop recycling technology, regulatory frameworks	Mid- to Long-term	[40]

### 2.2. Resource-based view

The RBV provides a theoretical framework to explain performance differences among firms in the same industry [41]. Such disparities in firm performance primarily arise from the heterogeneity of intangible resources [42]. Resources are key factors in a firm’s competitive advantage and must be valuable, inimitable, scarce, and non-substitutable [43]. Empirical studies have identified resources across various industries (Table 2) and clarified the causal relationships between key factors required for creating resource-based competitive advantages and performance [34]. Essentially, a firm’s capabilities and competitive advantage are closely linked to its resources [35]. Therefore, firms embark on strategic development of new resources by utilizing existing resources to secure sustainable competitive advantages [58,59]. However, in certain environments, the mere presence of resources may not be sufficient to maintain a strong competitive advantage, necessitating the possession of key capabilities based on a combination of key resources and complementary factors [47].

**Table 2:** Key resources.

Category	Key resources and capabilities	Studies
**Intellectual resources**	R&D intensity	[44]
	Technological capability	[45]
	The age of available technology	[44]
	The level of firm’s technology	[44]
	Brand age, brand equity	[46]
	No. innovations products	[47]
**Human resources**	Ownership structure	[48]
	The leader’s entrepreneurial traits, uniqueness of human capital	[44]
	Level of quality of employees	[49]
	Qualified staff	[50]
	entrepreneurial orientation	[51]
**Organizational resources**	Network	[48]
	Supplier relationship capability	[48]
	Firm size	[48]
	Incorporation age	[52]
	ESG disclosures scores	[53]
	Marketing skills	[54]
	CSR(Corporate Social Responsibility) intensity	[55]
**Financial resources**	Asset, debt, equity, Finances	[56]
**Physical resources**	Equipment, facilities	[57]

RBV has been widely applied in various industries to identify key resources that create sustainable competitive advantages. In the EV industry, key resources initially revolved around battery technology, cost reduction strategies, and supply chain management [60]. However, as competition intensified, the set of critical resources evolved to include brand equity, end-to-end integration capabilities, regulatory adaptability, and global production networks.

Empirical research highlights that firms must combine tangible and intangible resources to maximize market impact. While battery advancements and supply chain efficiency play direct roles in reducing costs, strategic R&D investments, regulatory adaptation, and ecosystem partnerships are necessary for long-term market expansion. Table 2 summarizes key RBV-based competitive resources in the EV industry.

Despite extensive studies on EV adoption factors, a research gap remains in understanding how firms strategically combine these resources across different market stages. Prior studies have analyzed consumer-level adoption but have not sufficiently explored firm-level resource interactions under TOE and RBV frameworks. This study addresses this gap by examining the evolving combinations of key resources and their impact on mid- to long-term EV market diffusion.

By integrating TOE and RBV, this study provides a comprehensive analysis of how firms leverage both external market conditions and internal capabilities to achieve sustainable EV market growth. This approach enhances existing research by identifying minimum threshold levels of key resources required for success at different market diffusion stages.

## 3. Methodology

### 3.1. Research framework

**Fig 1:**
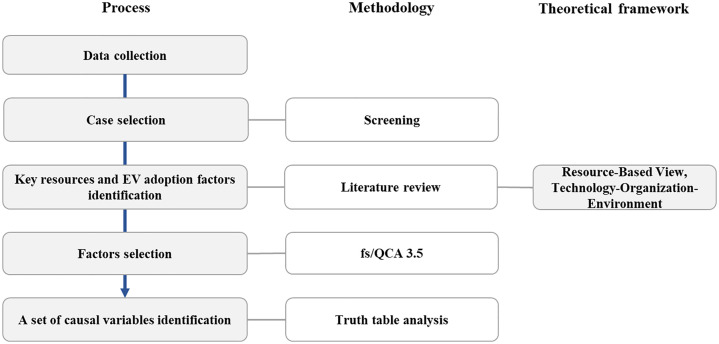
Research framework.

This study aims to identify the combinations of key resources and growth factors necessary for enabling mid- to long-term diffusion and growth in the EV market, and to estimate the minimum threshold for success. As shown in Fig 1, data on companies launching EVs is collected for the years 2012–2021. Data from companies that did not attempt market entry or have missing, or unreliable resource data are excluded. Then, based on a literature review, candidates for key resources and mid- to long-term growth factors are selected, and causal and outcome variables are defined.

Based on a comprehensive literature review, potential key resources and mid- to long-term growth factors are identified, leading to the definition of causal and outcome variables. This study is grounded in the Resource-Based View (RBV) and the Technology-Organization-Environment (TOE) framework, which provide the theoretical foundation for understanding the interplay between strategic firm-level resources and external environmental conditions in driving long-term EV market diffusion and firm performance.

To achieve robust analytical outcomes, our model must meet two critical requirements: 1)Minimizing bias associated with small sample size limitations, and 2)Identifying complex interactions among multiple causal variables. fs/QCA is chosen because it meets both requirements [61]. Following this, the causal and outcome variables are transformed into fuzzy scores through a calibration step, and fs/QCA analysis is conducted using fs/QCA 3.5 software. A truth table is generated that provides a set of causal variables representing key resources for entering the EV market.

This structured approach enables us to evaluate the dynamic interplay between internal firm resources and external market conditions, providing insights into which resource combinations drive sustained market growth and how firms can optimize their strategic positioning in the evolving EV landscape.

### 3.2. Data collection

In the five years from 2017 to 2022, EV sales surged from about 1 million to over 10 million units. Considering the overall growth of the EV market, the data collection period was set from 2017 to 2022. in the five years from 2017 to 2022, sales surged from about 1 million to over 10 million units. Moreover, the sales volume in 2017 represented an increase of more than 57% in compound annual growth rate compared to 2016 [39].

EV data were primarily collected from the U.S. Department of Energy, International Energy Agency, corporate websites, and http://ev-database.org. There are 12 companies that launched EVs from 2017 to 2022. Since the focus is on the success of mid- to long-term growth in the EV market, companies attempting growth were selected. Owing to insufficient data, all Chinese EV companies were excluded. Notably, most of these companies have only entered their domestic markets. Therefore, a separate analysis is required for Chinese EV companies, in contrast to North American and European firms targeting the global market. Additionally, trucks, vans, and demonstration vehicles were excluded owing to their limited release in specific countries and resulting insufficient data.

### 3.3. Fuzzy-set qualitative comparative analysis(fs/QCA)

fs/QCA offers a set-theoretic approach to elucidate causal relationships between conditions and outcomes. It allows identification of conjectural causal relationships in which combinations of conditions jointly lead to an outcome and explores the presence of specific conditions in cases related to the occurrence of an outcome [62]. Additionally, it reveals equifinality, in which multiple combinations of causal variables can produce the same outcome. As evidenced in the application to 17 cases in this study, its value is proven even in small-scale data. Owing to these advantages, fs/QCA is extensively used in various fields including business, sociology, and economics [63].

When applying fs/QCA, the process includes listing all cases and converting causal and outcome variables into fuzzy membership scores ranging from 0 to 1 through calibration. Notably, the calibration origin is descending, and the median serves as the crossover point [64]. Subsequently, all possible inferences between causal and outcome variables are reviewed, and in truth table analysis, Boolean algebra is used to reduce the number of inferences supported by the data, with the number of possible combinations of causal variables being 2k if there are k causal variables. Combinations are selected based on specific threshold values for consistency and frequency using Boolean algebra. The result of this process generates solutions representing combinations of causal and outcome variables supported across multiple cases, and these solutions are classified into complex, parsimonious, and intermediate solutions.

## 4. Empirical analysis

### 4.1. Background

EV market has seen rapid growth over the past decade, and its future prospects are viewed positively [65]. Companies are also striving to identify and secure key factors influencing mid- to long-term growth and expansion [66]. Some companies like Tesla have successfully entered the market, but others have failed in their attempts to enter the EV market. Additionally, some companies have had to withdraw from the market even after successful entry. These companies failed to properly understand evolving key factors and thus could not secure the necessary resources. This phenomenon underscores the notion that the determinants of success in the mid- to long-term growth phase may differ from those required during the market entry phase. Furthermore, market expansion or contraction is rarely the result of a single cause, making it necessary to conduct a comprehensive analysis of the complex interactions among various contributing factors.

### 4.2. Data

To collect data on EV companies, it is necessary to establish criteria to classify these companies into similar groups and compare them. This study systematically categorized the companies into target market segments using various factors such as vehicle purpose, size, and engine type. As shown in Table 3, this study divided 17 vehicle types into six market segments. These vehicle types are geographically diverse, with three from American companies, seven from European companies, and two from Asian companies. The market segment criteria were used to fairly select representative EV models for each company and to compare their prices with ICEVs.

**Table 3:** EV Models and companies for each market segment.

Market segments	Model	Manufacturer	Release year	Marque origin
**A**	Fiat 500e	Stellantis	2013	Italy
	smart EQ forfour	Daimler AG	2017	Germany
**B**	BMW i3	BMW	2013	Germany
	Chevrolet Bolt EV	General Motors	2016	United States
	Honda e	Honda	2020	Japan
**C**	Hyundai Ioniq 5	Hyundai	2021	South Korea
	Nissan Leaf	Stellantis	2010	Japan
	Tesla Model 3	Tesla, Inc.	2017	United States
	Volkswagen ID.3	Volkswagen	2020	Germany
	Volkswagen ID.4	Volkswagen	2020	Germany
	Volvo XC40 Recharge	Volvo Cars	2020	Sweden
**D**	Audi Q4 e-tron	Audi	2021	Germany
	Ford Mustang Mach-E	Ford	2020	United States
	Tesla Model X	Tesla, Inc.	2015	United States
**E**	Jaguar I-Pace	Jaguar Land Rover	2018	United Kingdom
	Tesla Model S	Tesla, Inc.	2012	United States
**F**	Audi e-tron GT	Audi	2021	Germany

### 4.3. Resources and mid- to long-term adoption factors

Table 4 presents key resources and mid- to long-term growth factors for the primary EV models of 12 companies. The primary EV model for each company is defined as the model that demonstrates the highest sales performance within its segment. The process for selecting key resources is further elaborated in the following section.

**Table 4:** Key resources and mid- to long-term growth factors.

Model	Battery price ($/kWh)	Acceleration performance (0–100 sec)	Top speed (km/h)	Charging time (km/h)	ICV price to EV price ratio	Driving range (km)
**Audie-tronGT**	140	4.1	245	1030	1.181	405
**AudiQ4e-tron**	140	8.5	160	540	1.86	405
**BMWi3**	230	7.3	150	270	1.837	156
**ChevroletBoltEV**	200	7	145	188	1.181	520
**Fiat500e**	300	9	150	390	2.08	235
**FordMustangMach-E**	140	7	180	400	1.376	375
**Hondae**	140	8.3	145	180	1.576	170
**HyundaiIoniq5**	140	7.3	185	1020	1.532	390
**JaguarI-Pace**	200	4.8	200	360	1.728	380
**NissanLeaf**	400	6.9	157	340	1.197	235
**smartEQforfour**	150	12.7	130	22	1.386	160
**TeslaModel3**	150	4.4	233	750	1.383	405
**TeslaModelS**	150	3.8	249	563	2.631	560
**TeslaModelX**	150	4.6	249	467	2.999	455
**VolkswagenID.3**	140	7.9	160	570	1.322	350
**VolkswagenID.4**	140	8.5	160	520	1.387	285
**VolvoXC40Recharge**	140	7.4	160	540	1.919	345

In addition, this study collects data on company-specific key resources such as brand ranking, user convenience, and battery recycling initiatives (Table 5). Brand ranking is assessed using the average automotive brand rankings provided by Brand Finance from 2015 to 2021. User convenience is measured through the presence of end-to-end integration features, such as automatic seat adjustments, mirror positioning, and starter settings, which enhance the user experience. Battery recycling programs are evaluated based on the duration of a company’s active recycling initiatives. The implementation of battery recycling is particularly important for improving battery price competitiveness, reducing dependence on raw materials, and ensuring long-term sustainability in the EV sector.

**Table 5:** Company-Specific key resources.

Model	Auto brand ranking (average from 2015 to 2021)	End-to-end integration available	Battery recycling program operating period (year)
**Audie-tronGT**	8.7	O	4
**AudiQ4e-tron**	8.7	O	4
**BMWi3**	4	O	9
**ChevroletBoltEV**	11.3	X	1
**Fiat500e**	40.7	X	1
**FordMustangMach-E**	7.7	O	1
**Hondae**	6.3	O	0
**HyundaiIoniq5**	13.7	O	4
**JaguarI-Pace**	17	O	0
**NissanLeaf**	9.3	X	11
**smartEQforfour**	1.3	X	0
**TeslaModel3**	12	O	3
**TeslaModelS**	12	O	3
**TeslaModelX**	12	O	3
**VolkswagenID.3**	3	O	2
**VolkswagenID.4**	3	O	2
**VolvoXC40Recharge**	9.7	O	2

### 4.4. Definition of variables

#### 4.4.1. Outcome variable.

This study defines the outcome as a binary variable representing the success or failure of mid- to long-term growth in the EV market. Several proxy variables can be used, but total sales volume is the most commonly used one in previous research [67]. If the total sales volume did not reach a certain threshold in the market entry phase, companies invariably withdrew from the market. The most frequently used threshold is half of the maximum units sold [68]. Therefore, a normalized value of 0.5 is adopted as the threshold, and the total number of units sold is normalized between 0 and 1 [64].

#### 4.4.2. Resources and adoption factors.

This study identifies and categorizes the key determinants of mid- to long-term success in the electric vehicle (EV) market into two major domains:

- Key Resources (Resource → Sales): Firm-level internal assets and capabilities—such as battery technology, organizational structure, and technical integration—that directly contribute to market performance.
- Key Adoption Factors (Factors → Sales): Consumer- and environment-facing variables that mediate the influence of firm resources on sales, thereby shaping user adoption and long-term market growth.

To provide a systematic framework for interpreting these adoption factors, this study applies the Technology–Organization–Environment (TOE) model. Although some adoption factors such as driving range or charging time are also salient for consumer decision-making, they are classified here based on their origin—technological, organizational, or environmental—in alignment with prior studies [10].

The Technology-Organization-Environment (TOE) framework is applied to categorize the adoption factors as follows:

- Technological factors represent performance-driven features that originate in the engineering or design of EVs. These include:
- *Driving Range* (maximum distance per charge) [69],
- *Battery Charging Time* (time to full charge) [70],
- *Safety Features* (e.g., ADAS, crash performance) [71],
- *OTA Capability* (availability of Over-the-Air updates) [72],
- *Vehicle Performance* (acceleration, top speed) [38].
- Organizational factors reflect internal execution capacity, such as:
  - *End-to-End Integration* (coordination between production, R&D, and marketing) [38],
  - *Cost Structure* (manufacturing efficiency and economies of scale) [73],
  - *Human Resource Capacity* (skilled workforce for EV development) [74].
- Environmental factors include external market conditions such as:
  - *Government Incentives* (subsidies, grants) [65],
  - *Charging Infrastructure* (density and accessibility) [75],
  - *Regulatory Compliance* (adherence to policy) [39],
  - *Consumer Awareness* (public knowledge and perception) [76].

These categories are summarized in Table 6.

**Table 6:** Key Adoption Factors for Electric Vehicles Based on the TOE Framework.

Category	Adoption Factor	Description
Technological	Driving Range	Maximum distance per charge
	Battery Charging Time	Time required to fully charge the battery
	Safety Features	ADAS presence, crash test performance
	OTA Capability	Support for Over-the-Air software updates
	Vehicle Performance	Acceleration, top speed, and handling
Organizational	End-to-End Integration	Cross-functional coordination (R&D, production, marketing)
	Cost Structure	Production efficiency and economies of scale
	Human Resource Capacity	Availability of trained personnel for EV development
Environmental	Government Incentives	Tax benefits, purchase subsidies
	Charging Infrastructure	Availability and density of public charging stations
	Regulatory Compliance	Conformance to environmental and safety regulations
	Consumer Awareness	General public’s perception and knowledge about EVs

Due to the limitations in directly linking each resource with specific adoption factors—arising from multicollinearity and data granularity—this study follows a two-step analytical approach:

- Resource-to-Sales Analysis: Identifies which firm-level internal resources are most influential on sales performance.
- Adoption Factor Mediation: Analyzes how key adoption factors influence the strength and direction of resource impact on market outcomes.

This indirect approach enables a comprehensive understanding of how EV firms can strategically align internal capabilities with market-facing adoption drivers to enhance mid- to long-term success.

#### 4.4.3. Resources and their impact on sales.

Technological resources for mid- to long-term growth include the battery price per unit capacity, EV driving performance (acceleration and top speed), and charging time. Particularly, the battery price per unit capacity (T1; Table 7) is used as an indicator of battery efficiency and cost competitiveness, which constitutes a significant part of the cost structure of EVs [26]. A lower T1 indicates higher battery technological resources, EV cost competitiveness, and battery supplier capabilities.

**Table 7:** Definition of causal variables(Resources/Adoption Factors).

Group	Causal variables	Resources/Adoption Factors	Measurement objective
**Technical**	Battery price per unit capacity (T1)	Resource	Battery price competitiveness and production efficiency
	EV acceleration performance (T2-1), EV Top speed(T2-2)	Resource	Performance of the drive system linked to EV driving
	Charging time (km/h; T3)	Resource	EV charging system performance
**Sustainability**	Implementation of battery recycling by the company (S1)	Resource	EV battery reuse/recycling capability
**Market**	Ratio of EV price to ICEV price by segment (M1)	Adoption Factor	EV price competitiveness
	Driving range (km; M2)	Adoption Factor	Maximum driving range
	Company’s brand value (M3)	Adoption Factor	EV company brand competitiveness
	End-to-end integration (M4)	Adoption Factor	EV customer convenience features

Acceleration performance (T2-1) and top speed (T2-2) are indicators of EV driving performance. Technologically, this is related to the battery, electric motor, control systems, etc. Acceleration performance is a key purchasing factor for consumers choosing EVs, so improving it has a positive impact on the growth and dissemination of the EV market [30]. Lower values for T2-1 and higher values for T2-2 indicate better technological resources related to the vehicle’s drive system linked to driving performance.

With increasing customer demand and market demand for longer driving ranges, EV battery capacities are gradually increasing. Accordingly, fast charging performance is an important factor in consumer adoption of EVs. The lower T3 is, the closer it is to rapid charging, indicating better performance of the EV charging system.

The implementation of battery recycling by the company (S1) is an important factor from the sustainability aspect of EVs [71]. To achieve competitive pricing for EVs, battery price competitiveness is necessary, and the capability to reuse or recycle EV batteries is an alternative to achieve this. Moreover, implementing battery recycling is important for conserving natural resources and protecting the environment [77]. Many countries legally regulate the proper disposal and recycling of battery waste, so EV companies must maintain an adequate recycling system in the future.

#### 4.4.4. Adoption factors and their mediating role.

Previous studies considered the price of EVs (M1) and driving range (M2) as key factors in consumers’ choice of EVs [27]. While both factors are important for market entry, consumers are more sensitive to price (M1) than to driving range (M2), making price a more influential factor than driving range [72]. Consumers choose their preferred EVs by considering the trade-off between price (M1) and driving range [78].

The company’s brand value (M3) is a major factor in customers’ adoption of vehicles [40]. A company’s brand value is assessed based on the average brand ranking of the automotive companies. As it is an ordinal scale, a lower numerical value in the average ranking indicates higher brand value.

The provision of user convenience features (M4) is also a factor that encompasses both EV technology and the market [79]. Providing user convenience features tends to increase vehicle prices; hence, overall cost reduction in components is necessary to control this. Additionally, energy consumption increases with the inclusion of convenience features. Customers expect the same level of convenience features in EVs as offered in ICEVs. Especially as the number of companies and models entering the EV market continues to grow, consumers comprehensively consider other specifications in addition to the basic factors [80].

Some variables are not suitable for comparative analysis. For instance, tax benefits and incentives could be influenced by macroeconomic variables such as national household income levels and vary by country [21]. Moreover, charging infrastructure depends on factors like national energy independence and the national power grid and is not limited to specific companies or vehicle types [81]. Not controlling for these factors could introduce country-specific biases in the comparative analysis between companies. Maintenance and total cost of ownership are more useful for comparisons between EVs and ICEVs rather than between EVs, and there is not much difference among EVs in the same segment.

As shown in Table 8, this study uses a calibration method to convert raw data of EV model causal variables into fuzzy-set scores. This allows us to identify a mix of causal variables that can explain the success or failure of mid- to long-term EV market entry.

**Table 8:** Fuzzy scores of causal variables.

Model	Resources					Adoption Factors			
	T1	T2-1	T2-2	T3	S1	M1	M2	M3	M4
**Audie-tronGT**	0.95	0.94	0.95	0.95	1	0.95	0.62	0.56	1
**AudiQ4e-tron**	0.95	0.34	0.51	0.6	1	0.34	0.62	0.56	1
**BMWi3**	0.28	0.51	0.27	0.21	1	0.35	0.05	0.88	1
**ChevroletBoltEV**	0.35	0.56	0.18	0.13	1	0.95	0.91	0.45	0
**Fiat500e**	0.14	0.28	0.27	0.37	1	0.25	0.13	0.05	0
**FordMustangMach-E**	0.95	0.56	0.66	0.39	1	0.79	0.51	0.65	1
**Hondae**	0.95	0.36	0.18	0.13	0	0.48	0.06	0.75	1
**HyundaiIoniq5**	0.95	0.51	0.7	0.95	1	0.51	0.56	0.4	1
**JaguarI-Pace**	0.35	0.89	0.79	0.33	0	0.4	0.52	0.32	1
**NissanLeaf**	0.05	0.58	0.43	0.3	1	0.95	0.13	0.51	0
**smartEQforfour**	0.51	0.05	0.05	0.05	0	0.78	0.05	0.95	0
**TeslaModel3**	0.51	0.92	0.92	0.82	1	0.78	0.62	0.44	1
**TeslaModelS**	0.51	0.95	0.95	0.63	1	0.1	0.95	0.44	1
**TeslaModelX**	0.51	0.91	0.95	0.51	1	0.05	0.79	0.44	1
**VolkswagenID.3**	0.95	0.42	0.51	0.63	1	0.86	0.42	0.91	1
**VolkswagenID.4**	0.95	0.34	0.51	0.57	1	0.78	0.23	0.91	1
**VolvoXC40Recharge**	0.95	0.49	0.51	0.6	1	0.31	0.4	0.49	1

## 5. Results

The electric vehicle (EV) market has experienced rapid expansion, driven by technological advancements, regulatory support, and evolving consumer preferences. However, not all EV manufacturers achieve sustained market success, highlighting the need to understand the key resources and adoption factors that influence sales performance. This study employs fuzzy-set qualitative comparative analysis (fsQCA) to explore the causal relationship between key resources, adoption factors, and market success [82]. By examining various resource configurations, the study aims to identify the critical elements that contribute to successful EV adoption and long-term growth.

This research follows a two-stage fsQCA approach to analyze the impact of key resources on sales performance (Resource → Sales) and the mediating role of growth factors in this relationship (Factors → Sales). The analysis uncovers distinct causal configurations at each stage, shedding light on the determinants of EV market success and long-term growth.

In the first stage of the fsQCA, the study examines the direct influence of key resources on EV sales performance. Table 9 presents the causal configurations that determine market success or failure. Given the study’s limited sample size, a case count threshold of 2 and a consistency threshold of 0.7 were established in accordance with prior research. Consistency, defined as the proportion of causal configurations that reliably lead to the same outcome, serves as a measure of empirical support for the identified causal relationships. Higher consistency scores indicate stronger evidence supporting these relationships. This study provides valuable insights into the critical factors shaping the EV market and offers a framework for understanding the interplay between resources, adoption factors, and long-term success.

**Table 9:** Truth table(Analysis of Key Resources and Sales Performance).

Type	T1	T2-1	T2-2	T3	S	Cases (n)	Outcome	Cases	Consistency
**G1**	0	0	1	0	1	3	1 (success)	NissanLeaf(C), ChevroletBoltEV(B), BMWi3(B)	0.926531
**G2**	1	1	0	1	1	4	1 (success)	AudiQ4e-tron(D), VolvoXC40Recharge(C), VolkswagenID.3(C) (C), VolkswagenID.4(C)	0.928021

### 5.1. Analysis of resources and sales performance

The analysis reveals that five key resources significantly influence EV sales performance. Battery price competitiveness (T1) represents the cost efficiency of battery production, directly impacting overall vehicle affordability. Acceleration performance (T2-1) refers to a vehicle’s ability to increase speed quickly, a crucial factor for performance-oriented consumers. Top speed (T2-2) reflects the maximum velocity an EV can achieve, influencing consumer perceptions of vehicle capability. Charging speed (T3) determines how quickly a vehicle can be recharged, affecting usability and convenience. Finally, battery recycling support (S) represents sustainability initiatives aimed at reducing production costs and environmental impact [83].

Table 9 presents the fsQCA results, which identify two main causal configurations contributing to EV market success. Each configuration leverages a specific set of key resources to gain competitive advantage. This demonstrates two distinct strategic routes, tailored to varying consumer needs and market positioning.

### 5.1.1. Sustainability and Performance Balance: The G1 Configuration

The first configuration, G1, represents EVs that emphasize sustainability and balanced performance. Vehicles in this category do not focus on cost-efficient battery production or rapid acceleration but instead achieve market success through strong top-speed performance and sustainability initiatives. The absence of cost-efficient battery production in this configuration suggests that factors beyond affordability, such as brand positioning and eco-friendly strategies, play a crucial role in driving market success. Additionally, the fact that these models do not prioritize charging speed indicates that longer charging times do not necessarily hinder market adoption if the vehicle offers other compelling attributes.

This configuration is observed in Nissan Leaf, Chevrolet Bolt EV, and BMW i3, all of which have maintained a stable presence in the market despite their moderate acceleration and slower charging speeds. The findings suggest that consumers in this segment prioritize sustainability and top-speed capability over affordability and fast-charging convenience, making this a viable strategy for manufacturers targeting environmentally conscious buyers.

### 5.1.2. Cost efficiency and performance optimization: The G2 configuration

The second configuration, G2, highlights a performance-driven approach that emphasizes cost-efficient battery production, high acceleration, and fast charging speeds. In contrast to the first configuration, where top speed is a significant determinant of market success, this pathway does not prioritize maximum velocity as a crucial factor. Instead, acceleration and charging speed become primary competitive advantages, suggesting that consumers in this category value a combination of performance and convenience.

This configuration is evident in Audi Q4 e-tron, Volvo XC40 Recharge, Volkswagen ID.3, and Volkswagen ID.4, all of which have successfully positioned themselves in the EV market by optimizing cost-efficient battery production and high-performance driving capabilities. The results indicate that affordability, rapid acceleration, and fast charging contribute more to market success than top-speed performance in this category. Furthermore, sustainability remains a key factor in both configurations, reinforcing the industry’s growing emphasis on eco-friendly initiatives.

### 5.2. Analysis of adoption factors and sales performance

The analysis reveals that four key adoption-related growth factors significantly influence EV sales performance. Vehicle price competitiveness (M1) reflects the affordability of EVs compared to internal combustion engine vehicles (ICEVs), playing a crucial role in consumer purchasing decisions. Driving range (M2) determines the distance an EV can travel on a single charge, influencing consumer confidence in EV usability. Brand ranking (M3) represents the market positioning and reputation of the manufacturer, affecting consumer trust. End-to-end digital integration (M4) refers to software-based enhancements that improve user experience, including advanced infotainment systems and smart connectivity.

As shown in Table 10, the fsQCA results identify one dominant causal configuration (G4) that contributes to EV market success. This configuration relies on a distinct combination of key adoption factors to maximize market penetration and long-term sales growth.

**Table 10:** Truth table(Analysis of adoption Factors and Sales Performance).

Type	M1	M2	M3	M4	Cases (n)	Outcome	Cases	Consistency
**G3**	1	1		1	5	1 (success)	Audie-tronGT(D), TeslaModel3(C), VolkswagenID.3(C), VolkswagenID.4(C), HyundaiIoniq5(C)	0. 809069

Affordability, range, and digital integration: The G3 configuration.

The G4 configuration represents EVs that achieve market success by offering competitive pricing, extended driving range, and seamless digital integration. Notably, brand ranking (M3) is not a necessary factor for success, suggesting that consumers prioritize affordability, range, and digital enhancements over brand prestige when selecting an EV.

The absence of brand ranking as a dominant factor indicates that newer EV manufacturers can successfully compete with established brands if they provide an optimal balance of affordability and usability. Furthermore, the presence of extended driving range and digital integration highlights the growing consumer demand for EVs that offer both practical performance and a connected driving experience.

This configuration is observed in Audi e-tron GT, Tesla Model 3, Volkswagen ID.3, Volkswagen ID.4, and Hyundai Ioniq 5, all of which have effectively positioned themselves in the market by optimizing their pricing strategies, enhancing battery range, and integrating advanced digital technologies. The fsQCA results confirm a consistency score of 0.809069, indicating strong empirical support for this causal relationship.

### 5.3. Minimum threshold levels for EV market growth

The analysis conducted through fuzzy-set qualitative comparative analysis (fsQCA) successfully identifies the minimum threshold levels for successful EV market entries, establishing quantitative benchmarks for key resources and adoption factors that contribute to market growth. However, one notable limitation of the findings is the absence of clear threshold values for unsuccessful (failed) EV models. The fsQCA results did not generate causal configurations that exclusively represent failure cases, meaning that the study was unable to extract performance values that directly correlate with market failure. While it is possible to determine the minimum requirements for success, the analysis does not confirm whether failing to meet these thresholds necessarily results in commercial failure. This suggests that EV market failure is not dictated by rigid numerical cutoffs but rather by a more complex interplay of multiple conditions [84]. Although failure-specific configurations could not be identified, the fsQCA analysis successfully established the minimum performance thresholds for successful EV models. These thresholds indicate the lowest observed values among growth EVs, providing manufacturers with reference points for market viability.

Table 11 summarizes the minimum performance thresholds for key resources observed among successful EV models. Table 12 presents the minimum thresholds for adoption-related factors that support EV market growth [77].

**Table 11:** Minimum Performance Thresholds for Key Resources (Actual Values).

Resource Factor	Notation	Minimum Value Among Growth EVs	Interpretation for Market Success
**Battery Price ($/kWh)**	**T1**	**≤ 200** (Chevrolet Bolt EV, Jaguar I-Pace)	Battery cost must be **≤ $200/kWh** for competitive pricing.
**Acceleration Performance (0–100 km/h sec)**	**T2-1**	**≤ 8.5 sec** (Audi Q4 e-tron, Volkswagen ID.4)	Acceleration should be **≤ 8.5 sec** for mainstream adoption.
**Top Speed (km/h)**	**T2-2**	**≥ 160 km/h** (Audi Q4 e-tron, Volkswagen ID.4, Volvo XC40 Recharge)	Top speed should be at least **160 km/h** to remain competitive.
**Charging Speed (km/h)**	**T3**	**≥ 520 km/h** (Volkswagen ID.4, Volvo XC40 Recharge)	Charging speed must be **≥ 520 km/h** to reduce range anxiety.
**Battery Recycling Program Duration (Years)**	**S1**	**≥ 2 years** (Volkswagen ID.3, Volkswagen ID.4, Volvo XC40 Recharge)	Sustainability initiatives must operate for **at least 2 years** to contribute to long-term success.

**Table 12:** Minimum Performance Thresholds for Adoption Factors (Actual Values).

Adoption Factor	Notation	Minimum Value Among Growth EVs	Interpretation for Market Success
Vehicle Price Competitiveness (ICV Price to EV Price Ratio)	M1	≤ 1.919 (Volvo XC40 Recharge)	EV price must be ≤ 1.92 times the comparable ICEV price for affordability.
Driving Range (km)	M2	≥ 285 km (Volkswagen ID.4)	Driving range must be ≥ 285 km to meet consumer expectations.
Auto Brand Ranking (2015–2021 Average)	M3	≤ 13.7 (Hyundai Ioniq 5)	Strong brand recognition is not required, as brands with rankings as low as 13.7 have succeeded.
End-to-End Integration Available	M4	Yes (Required for all successful EVs)	Digital connectivity is a key adoption factor, present in all growth EVs.

## 6. Discussion

This study emphasizes that the long-term success of electric vehicles (EVs) extends beyond initial market entry factors such as cost competitiveness and government incentives. While affordability and subsidies played a crucial role in early EV adoption, sustainable market growth requires a more comprehensive approach that integrates technological advancements, strategic resource management, and evolving consumer expectations. These insights align with the Technology-Organization-Environment (TOE) framework, which explains how firms must navigate technological innovation, internal strategies, and external market conditions to establish a sustainable competitive advantage. Additionally, this study builds upon the Resource-Based View (RBV) by highlighting the importance of leveraging firm-specific resources to shape adoption factors and influence sales performance.

However, due to the complexity of direct resource-to-factor relationships and the limitations in data availability, this study adopted a structured analytical approach by focusing on how key resources impact sales performance and how key adoption factors mediate market expansion. While this study does not quantitatively analyze the causal link between resources and adoption factors, an examination of existing literature provides qualitative insights into their interactions.

The analysis of key resources in this study demonstrates that battery price competitiveness, acceleration performance, top speed, charging speed, and battery recycling support significantly influence EV sales performance. Battery price competitiveness (T1) is a fundamental driver of affordability, particularly in mass-market segments, as battery costs account for 30–40% of total EV production costs [26]. Cost-efficient battery production allows manufacturers to offer EVs at competitive prices relative to internal combustion engine vehicles (ICEVs), enhancing affordability and consumer adoption [26]. The TOE framework contextualizes this linkage by showing how technological advancements in battery chemistry, organizational efforts in supply chain optimization, and government subsidies collectively shape price competitiveness. This insight reinforces that reducing battery costs remains one of the most critical enablers of mass-market EV adoption.

High acceleration performance (T2-1) requires higher energy consumption, which can negatively impact driving range (M2) [78]. However, advancements in battery energy management systems (EMS) and regenerative braking technologies have enabled EVs to maintain high acceleration while mitigating range loss [85]. This suggests that balancing high performance with energy efficiency is crucial for EVs to appeal to both performance enthusiasts and mainstream consumers.

Similarly, previous studies indicate that top speed (T2-2) is closely linked to brand ranking (M3). High-performance EVs, such as the Tesla Model S Plaid and Lucid Air, have played a significant role in shaping brand reputation and increasing consumer perception of EV technology leadership. Unlike legacy automakers, new entrants in the EV market have leveraged performance branding as a competitive strategy to compensate for their lack of historical brand equity. From a TOE framework perspective, this relationship is driven by technological differentiation, organizational branding strategies, and shifting consumer perceptions. This suggests that new EV brands can establish credibility and competitiveness in the market by emphasizing top-speed and acceleration as key differentiators.

Literature supports a strong link between charging speed (T3) and driving range (M2). Fast-charging networks reduce range anxiety, allowing EVs with smaller battery packs to remain competitive [86]. For example, Tesla’s Supercharger network enables Tesla EVs to operate with smaller battery capacities than competitors while maintaining consumer confidence in usability. From a TOE perspective, this is enabled by technological progress (ultra-fast charging stations), organizational strategies (partnerships with charging networks), and government policies (infrastructure funding and incentives for fast-charging expansion). This indicates that improving charging infrastructure can help overcome driving range limitations and encourage broader EV adoption.

Additionally, prior research suggests that battery recycling (S1) plays a role in improving vehicle price competitiveness (M1). Circular economy strategies, such as battery recycling and second-life applications, can help reduce raw material dependency and lower production costs [87]. Battery sustainability initiatives contribute not only to environmental compliance but also to long-term EV market price stability. This underscores that investments in battery recycling programs can enhance EV affordability and price stability, making EVs more accessible to a broader range of consumers.

One of the key insights from this study is that safety, infrastructure, and cybersecurity are becoming increasingly important in shaping EV market competitiveness, particularly in relation to digital integration (M4). Government-mandated safety standards and crash test ratings are now critical adoption factors, as consumers prioritize vehicle security and reliability. High crash-test ratings and compliance with regulatory safety requirements significantly enhance consumer confidence and influence EV purchasing decisions. Additionally, automakers that integrate Advanced Driver Assistance Systems (ADAS), automated braking, and lane-keeping technologies gain a competitive edge by addressing safety concerns.

### 6.1. TOE framework mapping: Resource → Adoption Factor → Sales

As shown in Table 13, the relationships between resources (T), adoption factors (M), and market performance can be mapped within the TOE framework, illustrating how technological, organizational, and environmental factors interact in shaping EV adoption.

**Table 13:** Mapping resource-to-adoption factor relationships within the TOE framework.

Resource (T)	Adoption Factor (M)	TOE Mechanism	Sales Impact
Battery Price (T1)	Vehicle Price Competitiveness (M1)	Lower battery costs → Lower consumer prices	Higher affordability → Increased mass adoption
Acceleration (T2-1)	Driving Range (M2)	Optimized battery management balances performance and efficiency	Broader appeal beyond niche performance buyers
Top Speed (T2-2)	Brand Ranking (M3)	Performance branding attracts premium consumers	New entrants gain credibility, competing with legacy brands
Charging Speed (T3)	Driving Range (M2)	Fast-charging networks reduce range anxiety	Increased consumer confidence in EV usability
Battery Recycling (S1)	Vehicle Price Competitiveness (M1)	Circular economy models reduce material costs	Price-stable EV production, lower retail costs
Safety & Infrastructure (T)	Digital Integration (M4)	ADAS, OTA updates, and cybersecurity improve consumer trust	Higher adoption due to reliability and long-term security

### 6.2. Strategic and policy implications

From a strategic perspective, EV manufacturers should align their product development strategies with evolving market dynamics. While cost efficiency and driving range remain crucial for mass-market EVs, premium segments must prioritize performance, charging infrastructure compatibility, and digital ecosystem integration. Sustainability initiatives, once seen as optional, are now a competitive necessity. Additionally, manufacturers must recognize that traditional brand equity is no longer a primary determinant of consumer adoption. Instead, offering competitive pricing, sufficient range, and seamless digital connectivity plays a more significant role in influencing purchasing decisions.

From a policy perspective, governments should implement differentiated incentives to balance market growth across segments. Targeted infrastructure investments, digital standardization, and safety regulations can accelerate EV adoption while ensuring continued technological innovation.

From a managerial perspective, the findings indicate that successful EV manufacturers strategically align their technological investments, organizational adaptability, and responses to external market conditions. Key determinants of market entry and long-term competitiveness include battery price competitiveness, charging infrastructure development, performance optimization, digital integration, and sustainability initiatives. For policymakers, beyond purchase subsidies, long-term industry growth requires a focus on expanding charging networks, standardizing digital connectivity, supporting battery lifecycle management, and incentivizing domestic battery production. A balanced regulatory framework aligned with technological advancements can accelerate adoption while ensuring market stability.

In conclusion, this study reinforces that EV market success is driven by a complex interplay of technological, organizational, and environmental factors. Through strategic alignment with evolving industry trends, manufacturers and policymakers can accelerate the transition to electric mobility and ensure long-term market stability.

## 7. Conclusion

The electric vehicle (EV) market has undergone a significant transformation, requiring firms to adopt a strategic approach beyond cost efficiency and government incentives. This study, based on the Resource-Based View (RBV) and the Technology-Organization-Environment (TOE) framework, highlights that market competitiveness depends on a combination of technological capabilities, organizational strategies, and external environmental factors. Our findings suggest that EV success is contingent upon firms’ ability to leverage internal resources while adapting to industry-wide technological, regulatory, and consumer-driven shifts.

This study contributes to the understanding of EV market dynamics by emphasizing the role of internal resource configurations alongside external factors such as infrastructure and policy incentives. The TOE framework illustrates how firms must integrate technological advancements, organizational capabilities, and environmental conditions to achieve sustainable growth. Unlike previous studies that primarily focus on policy support and infrastructure, this research highlights the importance of resource optimization in shaping EV adoption.

Despite its contributions, this study has limitations. The relatively small sample size restricts the generalizability of findings, necessitating further research with broader datasets and alternative methodologies. Additionally, while this study suggests potential causal relationships between resources and market success, these links require further validation through qualitative and quantitative analysis. The focus on passenger EVs excludes commercial and heavy-duty electric vehicles, which may require different strategic considerations. Moreover, macroeconomic factors such as global supply chain disruptions, energy price volatility, and geopolitical influences were not directly examined but likely play a role in shaping market outcomes. Future research should explore emerging technologies such as solid-state batteries, bidirectional charging, and AI-driven mobility services, as well as how firms can optimize technology adoption, organizational flexibility, and environmental adaptation in the evolving EV landscape.

Given these complexities, future research should explore alternative analytical methods to gain a deeper understanding of EV market failures. Case studies of discontinued EV models could provide insights into whether their struggles were linked to technical limitations, pricing misalignment, or broader economic challenges. Survival analysis models could assess whether suboptimal characteristics, such as high pricing, limited driving range, or weak digital integration, correlate with shorter product lifespans. Comparative market studies could further contextualize how regional policy variations, infrastructure development, and external economic conditions influence EV adoption patterns. By integrating these alternative research approaches, future studies could offer a more nuanced perspective on the determinants of market failure in the EV industry.

As the EV industry evolves, the interplay between technology, organizational strategy, and policy will remain critical. This study reinforces that EV market success is not solely dictated by technical specifications but by firms’ ability to adapt to shifting industry dynamics, consumer expectations, and regulatory frameworks. Companies that integrate cost-efficient technologies, optimize performance, and enhance digital capabilities will be better positioned for long-term competitiveness. Similarly, governments must implement forward-looking policies that support infrastructure expansion, sustainability, and technological standardization. Moving forward, a holistic approach that combines technological innovation, strategic agility, and regulatory foresight will be essential for the continued expansion and maturation of the EV market. Aligning industry efforts with policy support and consumer needs will be key to ensuring a successful transition toward sustainable mobility.

## Supporting information

S1 Data Fuzzy scores used in the fsQCA. This dataset contains the fuzzy membership scores of causal and outcome variables used in the study.
(CSV)
